# Factors influencing treatment status of syphilis among pregnant women: a retrospective cohort study in Guangzhou, China

**DOI:** 10.1186/s12939-023-01866-x

**Published:** 2023-04-06

**Authors:** Huihui Liu, Niannian Chen, Weiming Tang, Songying Shen, Jia Yu, Huiyun Xiao, Xingwen Zou, Jianrong He, Joseph D. Tucker, Xiu Qiu

**Affiliations:** 1 Division of Birth Cohort Study, Guangzhou Women and Children’s Medical Center, Guangzhou Medical University, 9 Jinsui Road, Guangzhou, 510623 China; 2 Department of Women’s Health, Guangdong Provincial Key Clinical Specialty of Woman and Child Health, Guangzhou Women and Children’s Medical Center, Guangzhou Medical University, 9 Jinsui Road, Guangzhou, 510623 China; 3 Guangdong Provincial Clinical Research Center for Child Health, Guangzhou Women and Children’s Medical Center, Guangzhou Medical University, 9 Jinsui Road, Guangzhou, 510623 China; 4 Institute for Global Health & Infectious Diseases, University of North Carolina, Chapel Hill, NC 27599 USA; 5 Faculty of Infectious and Tropical Diseases, London School of Hygiene and Tropical Medicine, Keppel Street, London, WC1E 7HT UK; 6 Provincial Key Laboratory of Research in Structure Birth Defect Disease and Department of Pediatric Surgery, Guangzhou Women and Children’s Medical Center, Guangzhou Medical University, 9 Jinsui Road, Guangzhou, 510623 China

**Keywords:** Syphilis, Treatment, Risk factors, Pregnant women

## Abstract

**Background:**

Many syphilis infected pregnant women do not receive treatment, representing a major missed opportunity to reduce the risk of syphilis-related adverse pregnancy outcomes. This study explored correlates of treatment among pregnant women with syphilis in Guangzhou, China.

**Methods:**

Pregnant women with a diagnosis of syphilis in Guangzhou between January 2014 and December 2016 were included. Information of syphilis treatment and correlates were extracted from a comprehensive national case-reporting system. Multivariate logistic regression was used to identify the correlations between information on the demographic characteristics, previous history, clinical characteristics about current syphilis, information of diagnosing hospital, and receiving no treatment or inadequate treatment among syphilis-seropositive pregnant women. A causal mediation analysis was used to explore the potential mediating role of the timing of syphilis diagnosis in the correlates.

**Results:**

Among 1248 syphilis-seropositive pregnant women, 379 (30.4%) women received no treatment or inadequate treatment. Migrant pregnant women (adjusted OR = 1.83, 95% CI: 1.25–2.73), multiparous participants (adjusted OR = 3.68, 95% CI: 2.51–5.50), unmarried participants (adjusted OR = 3.21, 95% CI: 1.97–5.28) and unemployed participants (adjusted OR = 2.43, 95% CI: 1.41–4.39) were more likely to receive no treatment or inadequate treatment. Participants who with history of syphilis infection (adjusted OR = 0.59, 95% CI: 0.42–0.82) and with high school and higher education participants (adjusted OR = 0.69, 95% CI: 0.49–0.97) were less likely to receive untreated or inadequately treatment. And that the impact of all these factors (except for the migrants) on treatment status are fully mediated through the syphilis diagnosis time, with the direct effect of migrants that would have resulted in a higher rate of no or inadequate treatment (OR = 2.34, 95% CI: 1.08–5.32) was partially cancelled out by the syphilis diagnosis time.

**Conclusions:**

Pregnant women who were migrant without local residence and women with syphilis diagnosed at a later gestational age were more likely to slip through the cracks of the existing antenatal care system. More programs should focus on eliminating these gaps of residence-related health inequalities. This research highlights actionable elements for health services interventions that could increase syphilis treatment rates among pregnant women.

**Supplementary Information:**

The online version contains supplementary material available at 10.1186/s12939-023-01866-x.

## Background

Maternal syphilis is a health threat to both pregnant women and their children. Without treatment, syphilis during pregnancy may result in stillbirth, neonatal death, prematurity, low birth weight, and congenital syphilis [1, 2]. Approximately one million pregnant women are infected with syphilis each year, leading to over 350,000 births with adverse outcomes (including 200,000 stillbirths and neonatal deaths) worldwide [3–5]. Preventing maternal syphilis is one of the most cost-effective public health measures that can strengthen health systems by improving access to quality-assured prenatal screening and saving newborn lives [6].

Timely treatment of maternal syphilis with an intramuscular injection of penicillin can reliably prevent mother-to-child transmission and other syphilis-related adverse pregnancy outcomes [7]. However, many infected women do not receive testing or treatment in low- and middle-income countries [8]. In 2013, around 38% of pregnant women with syphilis in China received untreated [9], similar to treatment rates among pregnant women with syphilis in Brazil [10]. Meanwhile, nearly all pregnant women with syphilis receive treatment in the United Kingdom [11].

To reduce the incidence of mother-to-child syphilis transmission in China, the Ministry of Health integrated and standardized the prevention programs for mother-to-child transmission (iPMTCT) of HIV, syphilis, and HBV in 2010. The ministry initiated nationwide comprehensive services [12]. According to the iPMTCT program, an opt-out syphilis testing and treatment program for all women receiving antenatal care (ANC) was established [12]. Pregnant women undergoing initial antenatal care and before delivery are tested for syphilis using two types of serologic tests: treponemal and nontreponemal assays. Pregnant women who first test positive with the nontreponemal test are confirmed with the treponemal test; pregnant women who first test positive with the treponemal test are followed with the nontreponemal test. Discordant results between two types of serologic tests are resolved with a second confirmatory treponemal test. In this program, obstetric care providers collect detailed clinical information on the women diagnosed with syphilis and provide them free treatment and follow-up care until delivery. Although the program has been running for several years, many syphilis-seropositive pregnant women still do not receive timely treatment. We previously reported that one quarter of syphilis-seropositive pregnant women failed to receive any syphilis treatment during pregnancy in Guangzhou, a city with high rates of hospitalized deliveries and high-quality maternal services [13]. At present, few studies have investigated correlates of syphilis treatment among pregnant women in large Chinese cities.

This underlines the need for more research to better understand treatment of syphilis during pregnancy. The purpose of this study was to identify correlates of receiving no treatment or inadequate treatment among pregnant women with syphilis.

## Methods

### Study design and participants

This retrospective cohort study used data were extracted from the iPMTCT program in China [12]. As part of the iPMTCT program, an obstetric care provider collects and reports basic information and clinical features related to the syphilis diagnosis and obstetric history among all pregnant women newly diagnosed with syphilis. In addition, they, and provide free treatment and follow-up for the syphilis-infected woman until she gives birth.

**Fig. 1:**
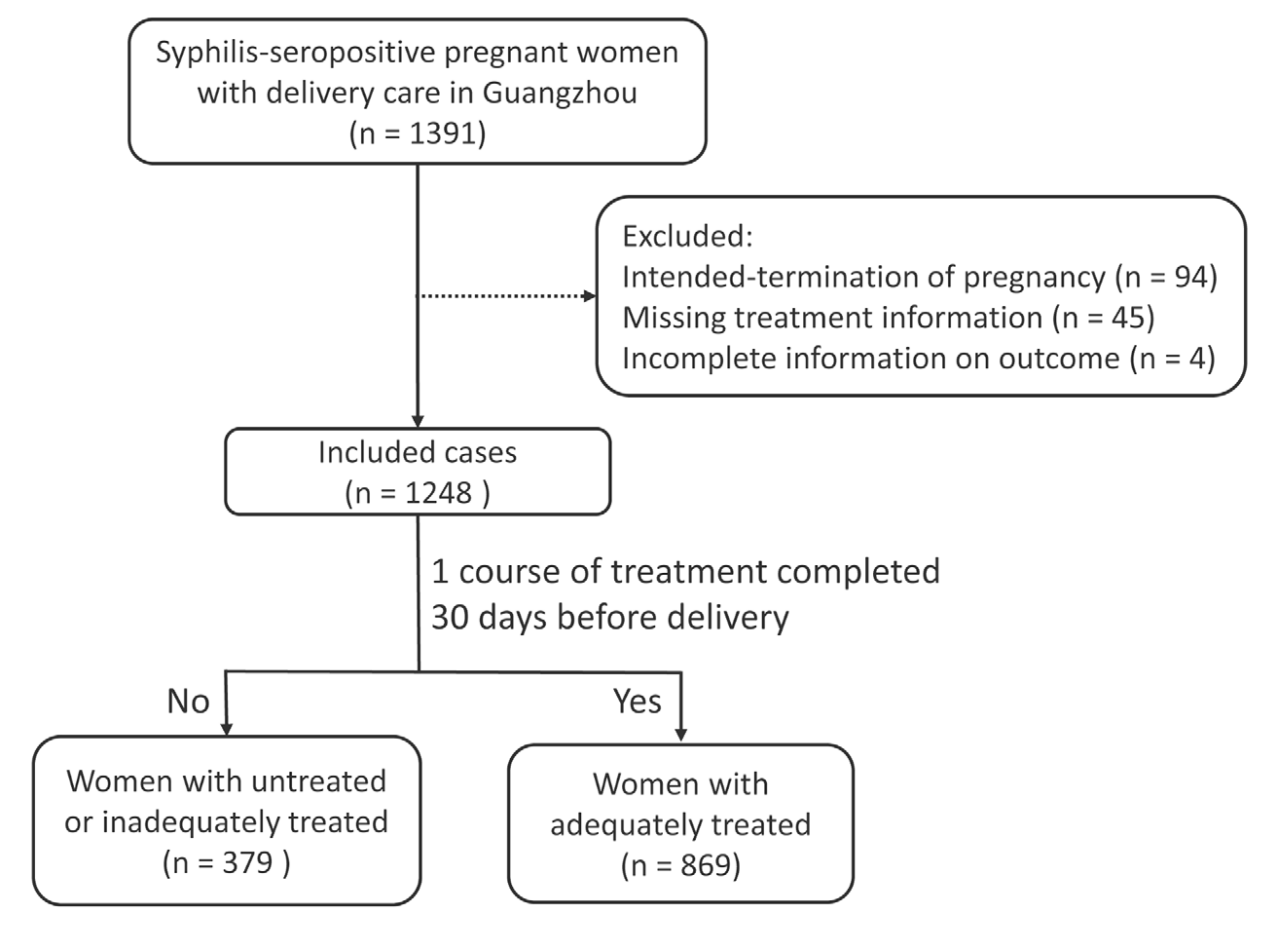
Flow chart of study participants

Between January 2014 and December 2016, 694,894 pregnant women hospitalized for delivery, of which 694,353 were screened for syphilis at delivery, and 635,546 were screened for syphilis during pregnancy. The present study included all syphilis-seropositive pregnant women who were reported to iPMTCT and received prenatal and delivery care in Guangzhou between January 2014 and December 2016 (n = 1,391). Participants who terminated their pregnancy (n = 94), had no treatment information (n = 45), or could not access complete information on outcome (n = 4) were excluded. As a result, a total of 1248 women were included in this analysis (Fig. 1). This study was approved by the Guangzhou Women and Children’s Medical Center Ethics Approval Board (reference number, 2,017,072,601).

### Outcomes

Syphilis infection was diagnosed by a positive treponemal serological test (either treponema pallidum particle agglutination assay (TPPA) or enzyme-linked immunosorbent assay (ELISA)), and a positive non-treponemal test (either toluidine red unheated serum test (TRUST) or rapid plasma reagin (RPR) test).

According to national guidelines[12], syphilis-seropositive pregnant women should be provided with free syphilis treatment for the prevention of mother-to-child transmission. Chinese guidelines recommend either benzathine penicillin 2.4 million units intramuscularly per week for three consecutive weeks or procaine penicillin 0.8 million units intramuscularly daily for 15 consecutive days. Ceftriaxone (one gram intravenous or intramuscularly daily for 10 consecutive days) may be used as an alternative to penicillin. For women who are allergic to penicillin, erythromycin (500 milligrams orally four times daily for 15 consecutive days) may be used. Full treatment must be initiated 30 days before delivery to prevent congenital syphilis.

The outcome was the treatment status during pregnancy defined as adequately treated and untreated or inadequately treated, adequate treatment as at least one course of treatment at least 30 days before delivery, and women who completed anything less than one course were defined as receiving no treatment or inadequate treatment.

### Exposure factors

Demographic and clinical characteristics of syphilis-seropositive pregnant women were collected by medical professionals using standardized questionnaires. (I) Demographic characteristics included age, education level (middle school or less, high school and higher), occupation (officer or business or service industry, farmer, and unemployed or others or unknown), marital status (first marriage, unmarried, and others). And residence was defined as local (with holding a local residence permit) and migrant (without holding a local residence permit). (II) Previous history included multipara (yes, no), history of adverse pregnancy outcome (yes, no), history of syphilis infection (yes, no). (III) Clinical characteristics on this syphilis infection included syphilis staging (latent, primary, secondary, tertiary), non-treponemal serum test titer (< 1:8 or ≥ 1:8), and gestational age at the time of syphilis diagnosis (< 28weeks or pre-pregnancy, ≥ 28weeks). Fourth, we also collected hospital information about hospital location (hospitals in Yuexiu, Haizhu, Liwan, Tianhe and Huangpu districts were categorized as urban; hospitals in Baiyun and Panyu districts were categorized as suburban; hospitals in Huadu, Conghua, Zengcheng & Nansha districts were categorized as rural), hospital administrative level (township, district, municipal/provincial), and type of diagnosing hospital (public hospital, private hospital).

We compared women with and without an adequately treated according to exposure factors status (age, education level, occupation, marital status, residence, multipara, history of adverse pregnancy outcome, history of syphilis infection, syphilis staging, non-treponemal serum test titer, gestational age at the time of syphilis diagnosis, hospital location, hospital administrative level, and type of diagnosing hospital).

### Statistical analysis

The screening rate analysis uses the number of all hospital births in Guangzhou, and the analysis of treatment factors is the number of pregnant women infected with syphilis in Guangzhou who were born or gave birth. Syphilis screening rate during pregnancy was calculated as the number of women in labor who had received syphilis tests during pregnancy divided by total number of women in labor. Syphilis screening rate at delivery was calculated as the number of women in labor who had received syphilis tests at delivery divided by total number of women in labor. The rate of untreated or inadequately treated syphilis was calculated as the number of pregnant women with untreated or inadequately treated syphilis during pregnancy divided by the total number of pregnant women with syphilis at delivery.

Descriptive analyses were used to describe the distributions in socio-demographic characteristics of study participants. T-tests were used to analyze continuous variables. Chi-square tests were used to summarize and compare categorical socio-demographic information and clinical characteristics participants with different treatment statuses and with different residence categories. Multivariable logistic regression models were used to analyze correlates of receiving no treatment or inadequate treatment among syphilis-seropositive pregnant women, all factors (age, residence, marital status, education, multipara status, history of adverse pregnancy outcome, history of syphilis infection, current staging of syphilis infection, non-treponemal serum test titer, location of diagnosing hospital, type of diagnosing hospital, and grade of diagnosing hospital) are entered into the model. When the analysis was restricted to women diagnosed with syphilis before 28 gestational weeks of pregnancy, the logistic regression model used a backward approach. We calculated odds ratios (OR) and 95% confidence intervals (CIs).

A causal mediation analysis [14] was used to explore the potential mediating role of time of syphilis diagnosis in the relationship between individual variables and receiving no treatment or inadequate treatment. The method allows for the decomposition of the total effect into direct and indirect (mediated through time of syphilis diagnose) components, while incorporating possible variable - time to syphilis diagnosis interactions. When the indirect effect was in the same direction as the total effect, the proportion (percentage) of the indirect effect was also calculated. We included time to syphilis diagnosis as a potential mediator and receiving untreated or inadequately treatment (1 = yes, 0 = no) as a separate outcome variable.

All analyses were conducted using SAS version 9.3 (SAS Institute, Cary, USA). Significance was set as α = 0.05.

## Results

### Syphilis screening in pregnant women

The syphilis screening rate during pregnancy increased between 2014 and 2016 (87.03%, 91.91%, 95.26). However, the syphilis screening rate at delivery remained above 99.9% (99.91%, 99.93%, 99.92%). The rate of receiving no or inadequate treatment for syphilis decreased from 35% to 2014 to 27% in 2016 (Fig. S1).

### Participant information

The demographic and clinical characteristics of the 1248 included participants are shown in Table 1. The mean age was 30.3 ± 6.0 years old. The majority of the participants were migrants (70.5%), married (79.5%), had middle school education level or below (54.6%), unemployed (51.9%), diagnosed in a public hospital (94.0%), and diagnosed with syphilis before 28 weeks gestation (67.2%). Overall, the rate of untreated or inadequately treated syphilis during pregnancy was 304 per 1000 syphilis-seropositive pregnant women (379/1248), and 82.1% of untreated or inadequately treated women were migrants. The percentage of untreated or inadequately treatment was higher in pregnant women who were migrants (35.3%), diagnosed at later gestational age (78.5%), with middle school or less education (35.1%), with higher parity (37.3%) and unmarried (50.0%) (Table 1). Among 1248 included participants, 81.2% of patients diagnosed before presentation for delivery, 18.8% of participants were diagnosed at or after delivery. Of 379 untreated or inadequately treated syphilis-seropositive pregnant women, 309 (81.5%) women did not any treatment for syphilis before delivery and 235 (62.0%) women were diagnosed at or after delivery.

**Table 1:** Baseline characteristics of syphilis-seropositive pregnant women with treatment or treatment failure during pregnancy in Guangzhou, China, 2014–2016 (N = 1248)

Variable	Women with untreated or inadequately treated N(%)	Women with adequately treated N(%)	Total N(%)	*P*-value^a^
Number	379 (30.4)	869 (69.6)	1248	
Age in years, mean ± SD	30.2 ± 6.4	30.4 ± 5.7	30.3 ± 6.0	0.488^b^
Residence				< 0.001
Local	68 (18.5)	300 (81.5)	368 (29.5)	
Migrant	311 (35.3)	569 (64.7)	880 (70.5)	
Marital status				< 0.001
First marriage	263 (26.5)	729 (73.5)	992 (79.5)	
Unmarried	71 (50.0)	71 (50.0)	142 (11.4)	
Others	45 (39.5)	69 (60.5)	114 (9.1)	
Education				< 0.001
Middle school or less	239 (35.1)	442 (64.9)	681 (54.6)	
High school and higher	100 (21.7)	362 (78.4)	462 (37.0)	
Unknown	40 (38.1)	65 (61.9)	105 (8.4)	
Occupation				< 0.001
Officer / Business / Service industry	25 (15.3)	138 (84.7)	163 (13.1)	
Farmer	41 (26.3)	115 (73.7)	156 (12.5)	
Unemployed or Others or Unknown	313 (33.7)	616 (66.3)	929 (74.4)	
Multipara				< 0.001
No	78 (17.9)	359 (82.2)	437 (35.0)	
Yes	300 (37.3)	505 (62.7)	805 (64.5)	
Unknown	1 (16.7)	5 (83.3)	6 (0.5)	
History of adverse pregnancy outcome				0.020
No	316 (31.9)	674 (68.1)	990 (79.3)	
Yes	63 (24.4)	195 (75.6)	258 (20.7)	
History of syphilis infection				< 0.001
No	267 (35.2)	492 (64.8)	759 (60.8)	
Yes	112 (22.9)	377 (77.1)	489 (39.2)	
Time of syphilis diagnose (gestational age, weeks)				
< 28w or pre-pregnancy	58 (6.9)	781 (93.1)	839 (67.2)	
≥ 28w	321 (78.5)	88 (21.5)	409 (32.8)	
Current staging of syphilis infection				0.125
Latent	302 (29.9)	708 (70.1)	1010 (80.9)	
Primary / Secondary / Tertiary	18 (24.0)	57 (76.0)	75 (6.0)	
Unknown	59 (36.2)	104 (63.8)	163 (13.1)	
Non-treponemal serum test titer				0.001
< 1:8	297 (28.4)	750 (71.6)	1047 (83.9)	
≥ 1:8	69 (39.0)	108 (61.0)	177 (14.2)	
Unknown	13 (54.2)	11 (45.8)	24 (1.9)	
Location of diagnosing hospital				0.002
Urban	107 (25.9)	306 (74.1)	413 (33.1)	
Suburban	157 (36.4)	274 (63.6)	431 (34.5)	
Rural	115 (28.5)	289 (71.5)	404 (32.4)	
Type of diagnosing hospital				< 0.001
Public	341 (29.1)	832 (70.9)	1173 (94.0)	
Private	38 (50.7)	37 (49.3)	75 (6.0)	
Grade of diagnosing hospital				< 0.001
Township	122 (40.7)	178 (59.3)	300 (24.0)	
District	103 (27.8)	267 (72.2)	370 (29.7)	
Municipal / Provincial	154 (26.6)	424 (73.4)	578 (46.3)	

Abbreviations: SD, standard deviation.

a: Chi-square tests; b: T-test;

### Factors influencing treatment of syphilis during pregnancy

The associations between participants characteristics and untreated or inadequately treatment were further explored via multivariable logistic regression. The results are shown in Table 2. Odds of untreated or inadequately treatment were higher among migrant participants (adjusted OR = 1.83, 95% CI: 1.25–2.73), multiparous participants (adjusted OR = 3.68, 95% CI: 2.51–5.50), unmarried participants (adjusted OR = 3.21, 95% CI: 1.97–5.28) and unemployed participants (adjusted OR = 2.43, 95% CI: 1.41–4.39). Participants who with history of syphilis infection (adjusted OR = 0.59, 95% CI: 0.42–0.82) and with high school and higher education participants (adjusted OR = 0.69, 95% CI: 0.49–0.97) were less likely to receive untreated or inadequately treatment. Pregnant women delivering across different location of diagnosing hospital (rural, suburban and urban areas) or different grade or different type of diagnosing hospital have similar rates of receiving untreated or inadequately treatment. The difference in rates of receiving untreated or inadequately treatment between the different current staging of syphilis infection and between the different non-treponemal serum test titers were not significant.

**Table 2:** Variables associated with receiving untreated or inadequately treatment for maternal syphilis

Variables	OR (95% CI)	*P*-value	Adjusted OR^a^ (95% CI)	*P-*value
Age	0.98(0.95-1.00)	0.086	0.99 (0.96–1.02)	0.410
Residence status				
Local	reference		reference	
Migrant	3.91 (2.60–6.09)	< 0.001	1.83 (1.25–2.73)	0.003
Marital status				
First marriage	reference		reference	
Unmarried	3.15(2.18–4.53)	< 0.001	3.21 (1.97–5.28)	< 0.001
Others	2.08(1.36–3.12)	0.001	1.26 (0.75–2.08)	0.372
Education				
Middle school or less	reference		reference	
High school and higher	0.48 (0.34–0.66)	< 0.001	0.69 (0.49–0.97)	0.033
Occupation				
Officer / Business / Service industry	reference		reference	
Farmer	2.23 (1.21–4.25)	0.012	1.74 (0.89–3.50)	0.109
Unemployed / Others / Unknown	3.15 (1.92–5.46)	< 0.001	2.43 (1.41–4.39)	0.002
Multipara				
No	reference		reference	
Yes	2.66 (1.87–3.83)	< 0.001	3.68 (2.51–5.50)	< 0.001
History of adverse pregnancy outcome				
No	reference		reference	
Yes	0.60(0.39–0.89)	0.014	0.66 (0.44–0.98)	0.045
History of syphilis infection				
No	reference		reference	
Yes	0.42 (0.30–0.59)	< 0.001	0.59 (0.42–0.82)	0.002
Current staging of syphilis infection				
Latent	reference		reference	
Primary / Secondary / Tertiary	0.74 (0.38–1.37)	0.363	1.15 (0.79–1.66)	0.478
Non-treponemal serum test titer				
< 1:8	reference		reference	
≥ 1:8	1.34 (0.94–1.89)	0.108	1.15(0.79–1.66)	0.477
Location of diagnosing hospital				
Urban	reference		reference	
Suburban	1.74 (1.20–2.55)	0.004	1.19 (0.81–1.76)	0.374
Rural	1.07 (0.72–1.58)	0.746	0.87 (0.58–1.30)	0.495
Type of diagnosing hospital				
Public	reference		reference	
Private	2.64 (1.47–4.74)	0.001	1.45 (0.74–2.85)	0.275
Grade of diagnosing hospital				
Township	reference		reference	
District	0.50 (0.33–0.75)	< 0.001	0.72 (0.46–1.13)	0.148
Municipal / Provincial	0.49 (0.34–0.71)	< 0.001	0.77 (0.51–1.18)	0.229

Abbreviations: OR odds ratio, CI confidence intervals

a: Adjusted for age, residence, marital status, education, multipara status, history of adverse pregnancy outcome, history of syphilis infection, current staging of syphilis infection, non-treponemal serum test titer, location of diagnosing hospital, type of diagnosing hospital, and grade of diagnosing hospital.

### The mediating role of syphilis diagnosis time

**Fig. 2:**
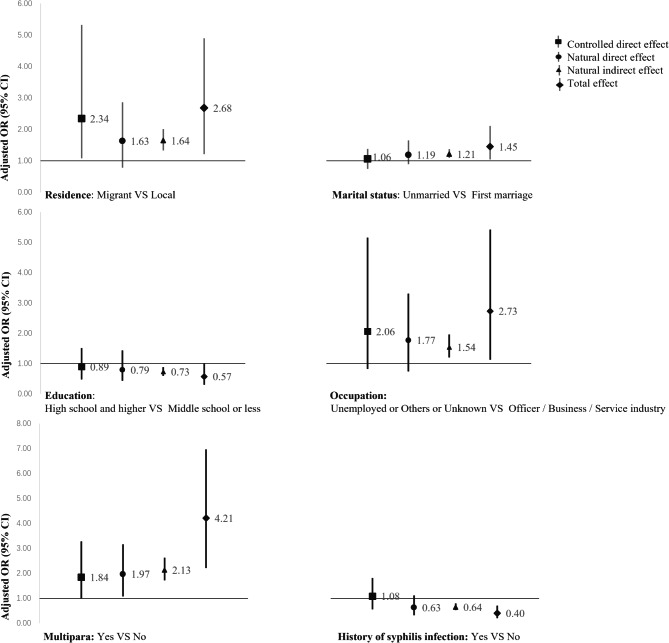
Mediation effect of syphilis diagnose time on the correlates of receiving nontreatment or inadequate treatment. Adjusted for residence, marital status, occupation, education status, multipara, history of syphilis infection. Abbreviations: *OR* odds ratio, *CI* confidence intervals

There is a close relationship between gestational age of syphilis diagnosis and inadequacy of treatment, because women diagnosed at delivery or less than 30 days before delivery cannot by definition receive adequate treatment. We found that 307 (81.0%) of the 379 untreated or inadequately treated patients were diagnosed within 30 days before delivery, and that the timing of diagnosis was probably the most direct factor affecting treatment. We therefore restricted the time of diagnosis to before 28 gestational weeks for the analysis, and by backward, the final model retained the inclusion of only one factor - residence (OR = 5.42, 95% CI: 2.13–18.33). A mediated analysis of the timing of syphilis diagnosis during pregnancy was carried out. The indirect effect is in the same direction as the total effect of untreated or inadequately treatment, indicating that the relationship between various factors and untreated or inadequately treatment is mediated by the time of diagnosis of syphilis, which we observed to be significantly mediated by the timing of syphilis diagnosis. (Fig. 2) The positive association between residence status and untreated or inadequately treated (total effect) was partially mediated by the proportion of total effect mediated by the indirect effect of time to syphilis diagnosis, which was 62.6%, with a direct effect is greater than 1 (OR = 2.34, 95% CI: 1.08–5.32). The positive association between the marital status, multipara, occupation and untreated or inadequately treatment (total effect) was fully mediated by the indirect effect of syphilis diagnosis time, the direct effect being close to 1. The negative association between the education, history of syphilis infection and untreated or inadequately treatment (total effect) was entirely mediated by the indirect effect of syphilis diagnosis time, with a direct effect also close to 1 (OR = 0.89, 95% CI: 0.47–1.51 and OR = 1.08, 95% CI: 0.55–1.81 respectively).

### Factors related to treatment status for syphilis in pregnancy for migrants

Socio-demographic and clinical characteristics were compared between local and migrant participants (Table S1). Differences between local and migrant participants were observed in marital status, education, occupation, history of syphilis infection, time of syphilis diagnose, location/type, and grade of diagnosing hospital (all *P* < 0.05). This includes migrant women who are more likely to be diagnosed at ≥ 28 weeks of gestation and in private hospitals, etc.

Among local participants, multiparous women (adjusted OR = 5.69, 95% CI: 2.20–17.15), and women’s occupation unknown (adjusted OR = 7.02, 95% CI: 1.86–46.42) were more likely to receiving untreated or inadequately treatment. As an exception, a marginally significant lower was observed between without or with history of syphilis infection (adjusted OR = 0.39, 95% CI: 0.18–0.79). Among migrant participants, odds of receiving untreated or inadequately treatment were higher among multiparous participants (adjusted OR = 3.40, 95% CI: 2.24–5.28), unmarried participants (adjusted OR = 3.30, 95% CI: 1.95–5.66) and unemployed participants (adjusted OR = 1.98, 95% CI: 1.09–3.80). Participants who with history of syphilis infection (adjusted OR = 0.66, 95% CI: 0.45–0.96) and with high school and higher education participants (adjusted OR = 0.62, 95% CI: 0.42–0.91) were less likely to receive untreated or inadequately treatment. (Table S2)

**Fig. 3:**
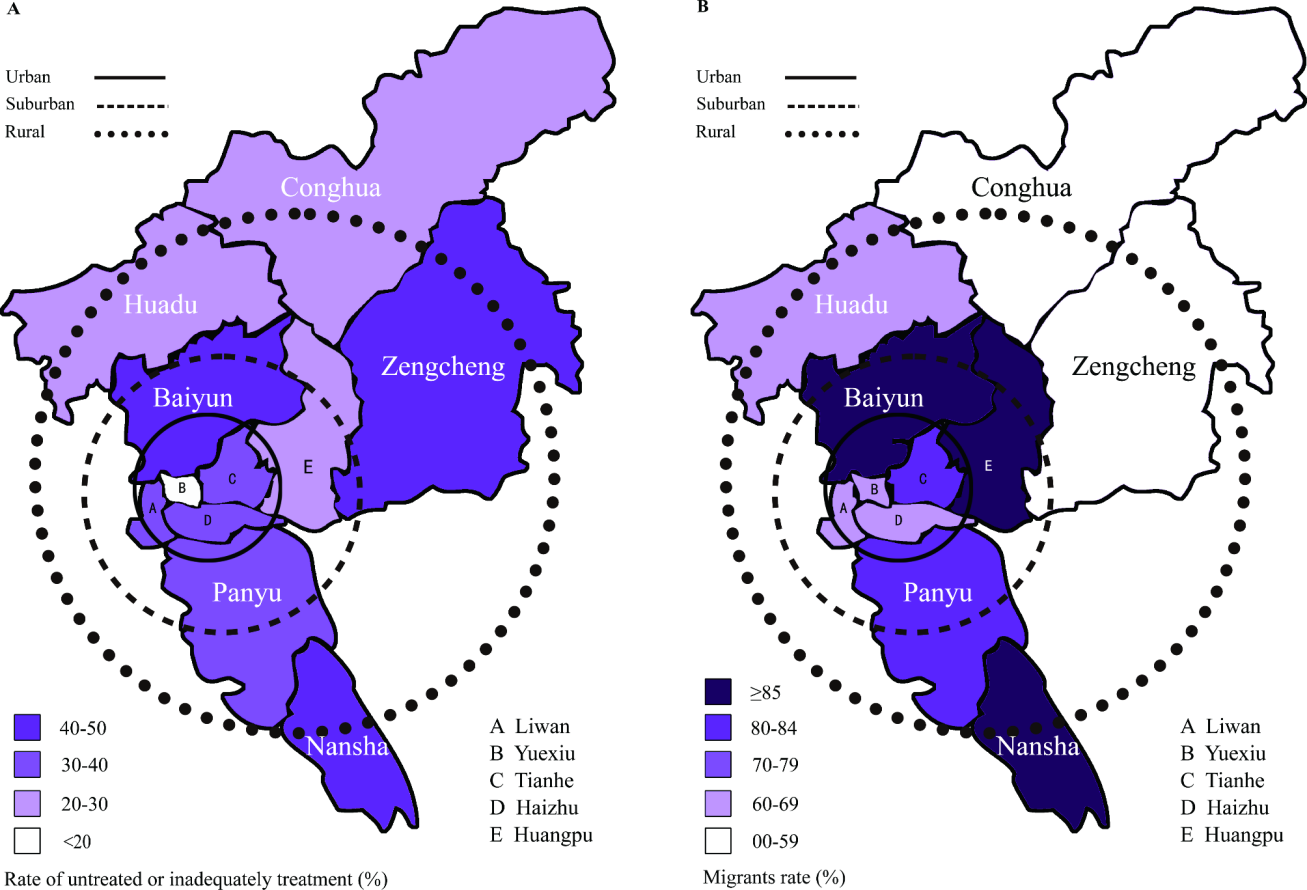
Rate of untreated or inadequately treated syphilis and the proportion of migrants in women infected with syphilis during pregnancy by districts. **A.** Rate of untreated or inadequately treated syphilis during pregnancy in pregnant women by districts. **B.** The proportion of migrants in women infected with syphilis during pregnancy by districts. The circles were not line up with geographical boundaries

As shown in A of Fig. 3 shows the rate of untreated or inadequately treated syphilis during pregnancy in pregnant women in 11 districts of Guangzhou. The rates of untreated or inadequately treated syphilis were lower in districts of Yuexiu, Huadu and Conghua, and vary among urban, suburban and rural hospitals (25.91%, 36.43%, 28.47%, *p* = 0.0024). B shows the proportion of migrants infected with syphilis during pregnancy in 11 districts of Guangzhou. These districts (i.e. Baiyun, Huangpu & Nansha) appears to be with a higher proportion of migrants infected with syphilis.

## Discussion

This study examined correlates of syphilis treatment among pregnant women in Guangzhou. We found that women who were migrants, multiparous, unmarried, unemployed, with low educational attainment and without history of syphilis infection were more likely to receive no or inadequate treatment, and that the impact of all these factors (except for the migrants) on treatment status are fully mediated through the timing of syphilis diagnosis. Furthermore, not receiving treatment or inadequate treatment was not significantly associated with hospital and current syphilis characteristics. Additionally, we found the syphilis screening rate increased and the rate of untreated or inadequately treated syphilis during pregnancy decreased over time.

We observed that one third of pregnant women with syphilis in Guangzhou did receive no treatment or inadequate treatment. This may be related to delayed screening, as multiple factors lead to non-treatment due to syphilis being diagnosed later than 28 weeks of pregnancy. Delayed or absent antenatal care was common among women with syphilis. Around 56% of women with maternal syphilis were identified during pregnancy and more than 40% were diagnosed at delivery or postpartum period [9]. This suggests an opportunity for earlier syphilis screening in pregnancy.

Residence status can affect treatment status partly independently of the time of syphilis diagnosis, leading to non-treatment. Our data suggest migrant women were more likely to be diagnosed ≥ 28 weeks of gestation. This may reflect migrant women have delayed syphilis screening compared to local pregnant women. In China, the residence permit system is usually associated with local health care and social security [15]. There is a correlation between residence permit and health inequality [16, 17]. However, most pregnant women infected with syphilis are without holding a local residence permit in Guangzhou [13]. Many internal migrant women in China do not receive adequate prenatal care. China has a large domestic migrant population and a Chinese study examined their use of antenatal care and the factors influencing this using data from a large national survey of the migrant population. This study examined demographics and migration experiences as predictors of early pregnancy antenatal care and adequate antenatal visits. It was found that 12.6% of migrant women reported no check-ups in the first trimester, 27.6% had fewer than five antenatal visits during their most recent pregnancy and 12.3% had three or fewer prenatal visits, and demographic predictors of delayed and inadequate care included lower levels of education, lower income and lack of maternity insurance. [18] Migrant women with syphilis had less education and higher levels of unemployment. However, in addition to the late diagnosis of syphilis, there may be other peculiarities in migrant women that lead to their inadequate treatment. Policy implementation and supervision may need to be strengthened to guarantee syphilis screening at the first prenatal visit.

Most pregnant women infected with syphilis are migrants in Guangzhou [13], increasing the importance of understanding syphilis services for both temporary migrants, permanent migrants, and other transient people. In May 2019, a global action plan was agreed at the 72nd World Health Assembly, which seeks to establish a “framework of priorities and guiding principles… to promote the health of refugees and migrants”. The plan includes short- and long-term steps to mainstream migrant health care; enhance partnerships; strengthen health monitoring and information systems and counter misperceptions about migrant health [19]. With the continuous development of reform and opening up and economy, the number of migrants in Guangzhou has increased rapidly, reaching 5.52 million, accounting for about one third of the total population [20]. There may be a need for policy or advocacy for earlier syphilis screening and for precise management of treatment for non-local residence permit migrant women with syphilis.

Our data also suggest that migrant syphilis-seropositive pregnant women were more likely to be diagnosed in a private hospital. Previous literature shows that pregnant women attending private ANC clinics receive fewer STD services [21]. Among the 6% of syphilis infected pregnant women seeking ANC in private hospitals, 50.7% received no or inadequate treatment. However, we did not find that participants diagnosed in private hospitals were more likely to be untreated or inadequately treated syphilis. Given the current expansion of private healthcare and increasing government investment in primary care in China, more women may receive prenatal care at private hospitals in the coming years [22]. Comprehensive strategies to promote high-quality prenatal services may also needed in private hospitals.

Maternity and marital status can affect treatment status leading to non-treatment. Previous studies have indicated that grand multipara women were received lesser antenatal care, and had more adverse pregnancy compared to the primipara [23]. One Portuguese study aim to evaluate the adequacy of prenatal care according to the family structure (single motherhood vs. two-parent family) in a population with free universal access to prenatal care [24]. It was based on 8001 mothers from the Portuguese birth cohort Geração XXI, and a questionnaire was administered to women between 24 and 72 h after delivery at the public maternity hospital in Porto, Portugal, between April 2005 and August 2006. It was found that 5.5% (n = 438) were single mothers, that single mothers were more likely to have an unplanned pregnancy (OR = 4.55; 95% CI 1.02 to 1.76) and that the frequency of private antenatal care was significantly lower among single mothers (OR = 0.57; 95% CI 0.42 to 0.79) [24]. Perhaps more attention needs to be paid to these two groups in addition to the current implementation of the policy, with increased awareness or policy emphasis on the importance of maternity testing and syphilis treatment for them.

However, this study did not find that the grade, type, and region of the hospital caused individual syphilis treatment to be affected, and our screening rates have been consistently high, so potentially indicating that all hospital consultations are uniform and standardized, with no hospital variation. Also, the clinical information on syphilis at current diagnosis does not correlate with treatment status. Therefore, other factors may need to be explored to reduce the untreated rate.

This study has several limitations. First, the dataset for this study was obtained using the administrative systems and there is likely some misclassification bias [25]. Second, we only included data from a single large city, which is the provincial hub and has more resources and better infrastructure for screening and treating syphilis in pregnancy than most other settings in China. Syphilis-seropositive pregnant women who delivered outside of Guangzhou were not included, making our results an over-estimate of treatment rates. Additionally, we do not have access to the exact timing of the first prenatal visit. However, the guidelines state that STD testing should be performed at the first antenatal visit and delayed testing likely reflects delays in first prenatal visit. Our analysis could not differentiate whether pregnant women were diagnosed at the time of delivery or earlier in the pregnancy.

This study has several research and policy implications. Further implementation research is needed to improve screening and expand treatment. Quality improvement measures to enhance early syphilis testing among all women should be further investigated. Comprehensive strategies to promote high-quality prenatal services are needed in migrant women with non-local residence permit.

## Conclusion

Women without holding a local residence permit and women diagnosed later in pregnancy were less likely to receive syphilis treatment during pregnancy. Our data suggest many pregnant women slip through the cracks of the existing ANC system, providing actionable elements for health service interventions, and greater efforts are needed to eliminate disparities in syphilis treatment due to the residence permit system.

## Electronic supplementary material

Below is the link to the electronic supplementary material.

Supplementary Material Table S1 Baseline characteristics of syphilis-seropositive pregnant women with different residence in Guangzhou, China, 2014-2016 (N=1248)

Supplementary Material Table S2 Variables associated with receiving untreated or inadequately treatment for maternal syphilis in different residence status

Supplementary Material Fig. S1 Syphilis screening rate in pregnant women and syphilis prevalence in pregnant women

## Data Availability

The data that support the findings of this study are available from the Health and Family Planning Commission of Guangzhou Municipality. ( http://www.gzmed.gov.cn/rhingzmed/xxgk/xxgkzn/25769.html ) but restrictions apply to the availability of these data, which were used under license for the current study, and so are not publicly available. Data are however available from the authors upon reasonable request and with permission of the Health and Family Planning Commission of Guangzhou Municipality.

## Abbreviations

ANC: Antenatal Care
iPMTCT: Integrated Prevention of Mother-to-Child Transmission
SD: Standard deviation
OR: Odds ratio
CI: Confidence intervals
