# Supplementary material for: Factors influencing treatment status of syphilis among pregnant women: a retrospective cohort study in Guangzhou, China
Source: Int J Equity Health. 2023 Apr 6;22:63. doi: 10.1186/s12939-023-01866-x (PMC10080893; doi:10.1186/s12939-023-01866-x)
Supplement: Supplementary file 1 — Supplementary Material Table S1 Baseline characteristics of syphilis-seropositive pregnant women with different residence in Guangzhou, China, 2014-2016 (N=1248) [file 12939_2023_1866_MOESM1_ESM.docx]

| **Table S1** Baseline characteristics of syphilis-seropositive pregnant women with different residence in Guangzhou, China, 2014-2016 (N=1248) | | | | | | | |
| --- | --- | --- | --- | --- | --- | --- | --- |
|  | **Local** | |  | **Migrant** | |  |  |
| **Variable** | **N** | **(%)** |  | **N** | **(%)** |  | ***P^a^*** |
| Marital status |  |  |  |  |  |  |  |
| First marriage | 310 | (84.2) |  | 682 | (77.5) |  | 0.001 |
| Unmarried | 23 | (6.3) |  | 119 | (13.5) |  |  |
| Others | 35 | (9.5) |  | 79 | (9.0) |  |  |
| Education |  |  |  |  |  |  |  |
| Middle school or less | 172 | (46.7) |  | 509 | (57.8) |  | 0.002 |
| High school | 160 | (43.5) |  | 291 | (33.1) |  |  |
| College and higher | 5 | (1.4) |  | 6 | (0.7) |  |  |
| Unknown | 31 | (8.4) |  | 74 | (8.4) |  |  |
| Occupation |  |  |  |  |  |  |  |
| Officer / Business/ Service industry | 64 | (17.4) |  | 99 | (11.3) |  | < 0.001 |
| Farmer | 59 | (16.0) |  | 97 | (11.0) |  |  |
| Unemployed or Others or Unknown | 245 | (66.6) |  | 684 | (77.7) |  |  |
| Multipara |  |  |  |  |  |  |  |
| No | 134 | (36.4) |  | 303 | (34.4) |  | 0.644 |
| Yes | 233 | (63.3) |  | 572 | (65.0) |  |  |
| Unknown | 1 | (0.3) |  | 5 | (0.6) |  |  |
| History of adverse pregnancy outcome |  |  |  |  |  |  |  |
| No | 77 | (20.9) |  | 181 | (20.6) |  | 0.886 |
| Yes | 291 | (79.1) |  | 699 | (79.4) |  |  |
| History of syphilis infection |  |  |  |  |  |  |  |
| No | 186 | (50.5) |  | 573 | (65.1) |  | < 0.001 |
| Yes | 182 | (49.5) |  | 307 | (34.9) |  |  |
| Time of syphilis diagnose (gestational age, weeks) | |  |  |  |  |  |  |
| <28w or pre-pregnancy | 290 | (78.8) |  | 549 | (62.4) |  | < 0.001 |
| ≥28 | 78 | (21.2) |  | 331 | (37.6) |  |  |
| Non-treponemal serum test titer |  |  |  |  |  |  |  |
| < 1:8 | 316 | (85.9) |  | 731 | (83.1) |  | 0.438 |
| ≥1:8 | 45 | (12.2) |  | 132 | (15.0) |  |  |
| Unknown | 7 | (1.9) |  | 17 | (1.9) |  |  |
| Location of diagnosing hospital |  |  |  |  |  |  |  |
| Urban | 126 | (34.2) |  | 307 | (34.9) |  | < 0.001 |
| Suburban | 69 | (18.8) |  | 342 | (38.9) |  |  |
| Rural | 173 | (47.0) |  | 231 | (26.3) |  |  |
| Type of diagnosing hospital |  |  |  |  |  |  |  |
| Public | 365 | (99.2) |  | 808 | (91.8) |  | < 0.001 |
| Private | 3 | (0.8) |  | 72 | (8.2) |  |  |
| Grade of diagnosing hospital |  |  |  |  |  |  |  |
| Township | 41 | (13.7) |  | 259 | (86.3) |  | < 0.001 |
| District | 139 | (37.6) |  | 231 | (62.4) |  |  |
| Municipal / Provincial | 188 | (32.5) |  | 390 | (67.5) |  |  |
| ^a^: Chi-square test |  |  |  |  |  |  |  |
