# Supplementary material for: Factors influencing treatment status of syphilis among pregnant women: a retrospective cohort study in Guangzhou, China
Source: Int J Equity Health. 2023 Apr 6;22:63. doi: 10.1186/s12939-023-01866-x (PMC10080893; doi:10.1186/s12939-023-01866-x)
Supplement: Supplementary file 2 — Supplementary Material Table S2 Variables associated with receiving untreated or inadequately treatment for maternal syphilis in different residence status [file 12939_2023_1866_MOESM2_ESM.docx]

| **Table S2** Variables associated with receiving untreated or inadequately treatment for maternal syphilis in different residence status | | | | | |
| --- | --- | --- | --- | --- | --- |
|  | **Local** | |  | **Migrant** | |
| **Variables** | **Adjusted OR (95% CI)** | ***P-*value** | | **Adjusted OR (95% CI)** | ***P-*value** |
| Age | 1.01 (0.95 - 1.08) | 0.729 |  | 0.98 (0.95 - 1.02) | 0.304 |
| Marital status |  |  |  |  |  |
| First marriage | reference |  |  | reference |  |
| Unmarried | 1.78 (0.32 - 8.05) | 0.472 |  | 3.30 (1.95 - 5.66) | <0.001 |
| Others | 0.97 (0.28 - 2.88) | 0.963 |  | 1.36 (0.76 - 2.42) | 0.295 |
| Education |  |  |  |  |  |
| Middle school or less | reference |  |  | reference |  |
| High school and higher | 1.02 (0.48 - 2.18) | 0.962 |  | 0.62 (0.42 - 0.91) | 0.016 |
| Occupation |  |  |  |  |  |
| Officer / Business / Service industry | reference |  |  | reference |  |
| Farmer | 3.25 (0.66 - 24.15) | 0.180 |  | 1.58 (0.74 - 3.45) | 0.241 |
| Unemployed / Others / Unknown | 7.02 (1.86 - 46.42) | 0.013 |  | 1.98 (1.09 - 3.80) | 0.031 |
| Multipara |  |  |  |  |  |
| No | reference |  |  | reference |  |
| Yes | 5.69 (2.20 - 17.15) | 0.001 |  | 3.40 (2.24 - 5.28) | <0.001 |
| History of adverse pregnancy outcome | |  |  |  |  |
| No | reference |  |  | reference |  |
| Yes | 0.47 (0.16 - 1.19) | 0.136 |  | 0.73 (0.46 - 1.15) | 0.180 |
| History of syphilis infection |  |  |  |  |  |
| No | reference |  |  | reference |  |
| Yes | 0.39 (0.18 - 0.79) | 0.011 |  | 0.66 (0.45 - 0.96) | 0.033 |
| Current staging of syphilis infection | |  |  |  |  |
| Latent | reference |  |  | reference |  |
| Primary / Secondary / Tertiary | 0.57 (0.12 - 1.95) | 0.409 |  | 0.51 (0.23 - 1.04) | 0.073 |
| Non-treponemal serum test titer | |  |  |  |  |
| < 1:8 | reference |  |  | reference |  |
| ≥ 1:8 | 0.92 (0.35 - 2.27) | 0.860 |  | 1.21 (0.80 - 1.84) | 0.364 |
| Location of diagnosing hospital | |  |  |  |  |
| Urban | reference |  |  | reference |  |
| Suburban | 1.14 (0.38 - 3.28) | 0.810 |  | 1.15 (0.76 - 1.76) | 0.510 |
| Rural | 1.31 (0.56 - 3.17) | 0.543 |  | 0.75 (0.47 - 1.20) | 0.233 |
| Type of diagnosing hospital |  |  |  |  |  |
| Public |  |  |  | reference |  |
| Private | / |  |  | 1.64 (0.82 - 3.30) | 0.163 |
| Grade of diagnosing hospital |  |  |  |  |  |
| Township | reference |  |  | reference |  |
| District | 0.52 (0.16 - 1.83) | 0.289 |  | 0.76 (0.46 - 1.25) | 0.280 |
| Municipal / Provincial | 0.73 (0.24 - 2.47) | 0.595 |  | 0.76 (0.48 - 1.21) | 0.251 |
| Abbreviations: *OR* odds ratio, *CI* confidence intervals | | | |  |  |
