# Supplementary material for: Factors influencing treatment status of syphilis among pregnant women: a retrospective cohort study in Guangzhou, China
Source: Int J Equity Health. 2023 Apr 6;22:63. doi: 10.1186/s12939-023-01866-x (PMC10080893; doi:10.1186/s12939-023-01866-x)
Supplement: Supplementary file 3 — Supplementary Material Fig. S1 Syphilis screening rate in pregnant women and syphilis prevalence in pregnant women [file 12939_2023_1866_MOESM3_ESM.docx]

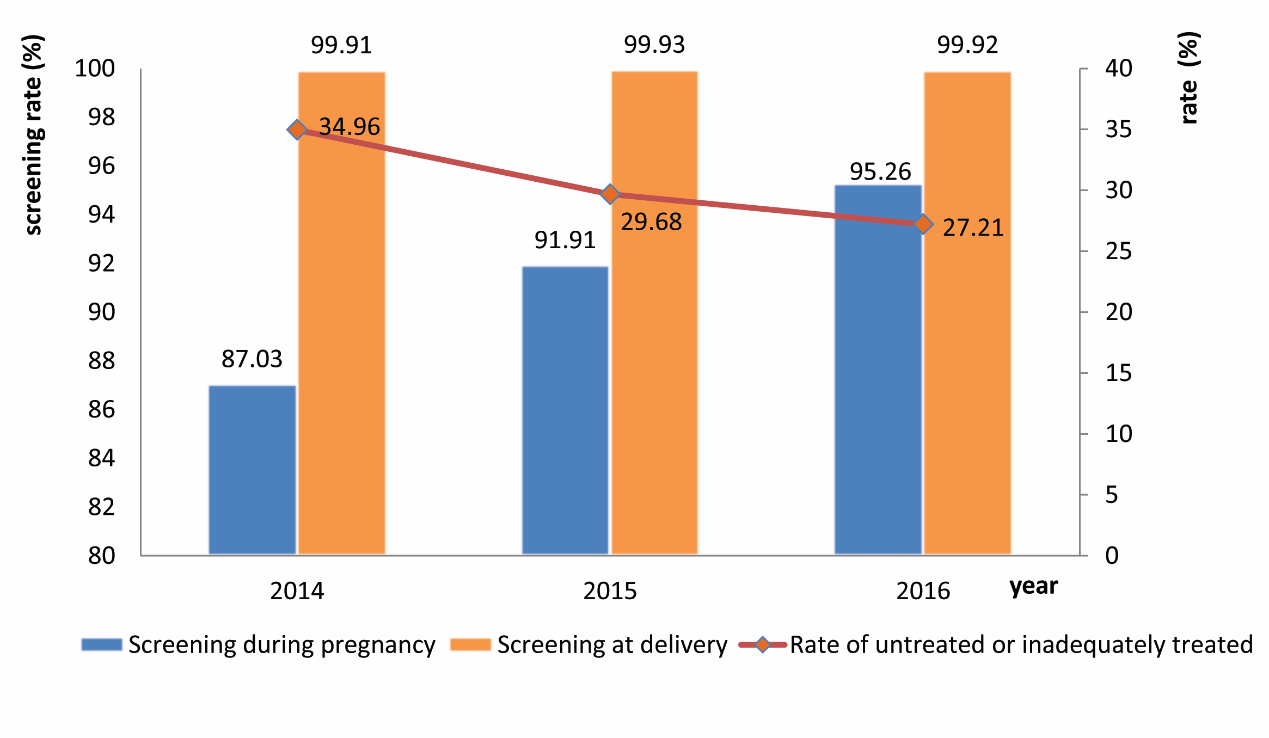


**Fig. S1** Syphilis screening rate in pregnant women and syphilis prevalence in pregnant women

Syphilis screening rate during pregnancy was calculated as the number of women in labor who had received syphilis tests during pregnancy divided by total number of women in labor. Syphilis screening rate at delivery was calculated as the number of women in labor who had received syphilis tests at delivery divided by total number of women in labor. The rate of untreated or inadequately treated syphilis was calculated as the number of pregnant women with untreated or inadequately treated syphilis during pregnancy divided by the total number of pregnant women with syphilis at delivery.
